# Post-Pandemic Evaluation of Emergency Medicine Workforce Stability

**DOI:** 10.26502/acmcr.96550751

**Published:** 2026-03-17

**Authors:** Fihr Chaudhary, Devendra K. Agrawal

**Affiliations:** Department of Translational Research, College of Osteopathic Medicine of the Pacific, Western University of Health Sciences, Pomona CA 91766, USA

**Keywords:** COVID-19, Emergency department, Emergency medicine, Physicians, Workforce stability

## Abstract

Physician workforce projections in emergency medicine were developed in the year 2020 based on the assumptions and baseline data during the unprecedented disruption in healthcare delivery due to COVID-19 pandemic. However, the fundamental assumptions in this project might not be realistic in the post COVID-19 era. In this article, the accuracy of 2020 emergency medicine workforce oversupply projection was critically evaluated and comprehensively reassessed considering post COVID-19 data. The findings support the underestimation of the demand and exaggeration of projected physician surplus. The post-pandemic trends suggest a more fragile emergency medicine workforce marked by sustained attrition and vulnerability to stressors. A stable or modest increase in the demand for emergency physicians between the years 2030 and 2040 is projected. The findings suggest that clinical workload per physician is likely to increase due to the emergency care of aging population, delayed presentations of acute and chronic diseases, and increased boarding and throughput challenges. Accordingly future workforce projections must incorporate updated attrition estimates and account for structural shifts in emergency care delivery. This would require maintaining or expanding demand for emergency physicians in next decade and beyond.

## Introduction

Physician workforce projections play a critical role in ensuring long-term access to emergency care. In 2020, a widely cited emergency medicine (EM) labor market forecast predicted a substantial surplus of board-certified emergency physicians by the year 2030. This projection carried both immediate and long-term consequences, influencing trainee career decision-making and shaping institutional strategies regarding residency expansion across the United States. However, the forecast was developed during a period of unprecedented disruption in health-care delivery caused by the COVID-19 pandemic. As such, concerns have emerged regarding the validity and durability of the assumptions and baseline data on which the projection was based. With the accumulation of post-pandemic data, it has become more apparent that some of the fundamental assumptions underlying the 2020 projection, such as consistent demand, minimal turnover, and the balancing of supply and demand, might not be very realistic representations of reality in the present or the future. Accordingly, a comprehensive reassessment of the projected EM workforce oversupply using post-pandemic evidence is warranted. Considering post-pandemic data, this article evaluated and reassessed the accuracy of the 2020 emergency medicine workforce oversupply projection.

## Methods

### Data Sources

The current analysis relied on several complementary sources of data as a way of capturing national and practice-level trends on the workforce in emergency medicine (EM). The publicly available databases used to get national workforce and training data were the Association of American Medical Colleges (AAMC), American Medical Association (AMA), National Resident Matching Program (NRMP) and the National Syndromic Surveillance Program (NSSP) [4–6]. This was based on the sources that were concerning the supply of physicians, residency placement, the output of training, and the use of emergency care. Staffing and recruitment patterns in ED were evaluated based on the national workforce report and institutional staffing data, comprising of surveys and reports of health systems, academic departments and commercially staffing large emergency medicine departments. The changes in the volume of emergency department visits, patient acuity and billing patterns were measured using administrative datasets over time. In addition, peer-reviewed literature and policy analyses published between 2018 and 2025 were systematically reviewed to inform a structured synthesis of emergency medicine workforce forecasting, pandemic-related workforce disruptions, and post-pandemic recovery trends.

### Analytical Approach

Comparative analyses were conducted to evaluate differences in workforce trends across three distinct periods: pre-pandemic (2018–2019), pandemic (2020–2021), and post-pandemic (2022–2025). This approach allowed for assessment of how pandemic-related deviations affected the assumptions underlying the 2020 workforce projection and their subsequent validity. Temporal trend analyses spanning 2018–2025 were performed to examine changes in workforce supply, demand, and patterns of practice. Key metrics included the ratio of practicing emergency physicians to emergency department visit volume, as well as trends in staffing models and scope-of-practice expansion. Workforce sustainability indicators, such as premature retirement and attrition, were incorporated to account for non-linear workforce losses that are inadequately captured by traditional forecasting models. Lastly, the effects of changing staffing models such as the rise of large corporate emergency medicine care on the workforce distribution, job opportunity, and flexibility of staffing were investigated using both qualitative and quantitative data. The combined analyses were then applied to determine whether the oversupply forecast used in 2020 was relevant to the present post-pandemic situation

## The 2020 Workforce Forecast: Overview and Limitations

### Summary of the 2020 Projection

The 2020 workforce analysis aimed to estimate the size of the emergency medicine workforce by 2030, with a primary focus on residency-trained, board-certified emergency physicians while also accounting for contributions from nurse practitioners and physician assistants. The model sought to project future supply and demand using contemporary workforce data combined with assumptions extrapolated from historical trends and anticipated developments. To predict the future supply and demand for workers, the study used a technique that included both current data sources and assumptions based on past trends and anticipated developments. A sensitivity analysis was used to investigate the potential impact of different baseline inputs and assumptions on the final prediction. In particular, the following procedures were employed: determining the present supply of practitioners in the workforce who are relevant (doctors, physician assistants, and nurse practitioners), making predictions about how the workforce will change in the future as a result of people joining and leaving the industry, examining the efficiency of the staff, forecasting the need for emergency medical assistance. This methodology looked at data from the base year, when supply and demand were equal, and monitored changes all the way up to 2030. According to the results, the best-case scenario for the future involves this: graduate medical education grows by 2% per year, a yearly attrition rate of 3% among emergency physicians, nurse practitioners and physician assistants are expected to handle 20% of patient encounters, and emergency department visits are expected to increase by 11% compared to 2018 [1]. Based on these projections, there would be 7,845 more emergency physicians than needed by 2030.

### Analysis of Limitations

#### Staffing was temporarily re-modelled during early COVID-19

The 2020 projection relied on the assumption of a stable market equilibrium, presuming that the contemporaneous supply of emergency physicians adequately met patient demand. While commonly employed in workforce modeling, this assumption may not have been valid in 2020.

Healthcare organizations around the world had to implement a range of unprecedented methods to effectively allocate resources and personnel in response to the high number of critically ill COVID-19 patients and the scarcity of health human resources (HHR) [2]. A comprehensive assessment revealed that the COVID-19 epidemic posed novel problems that called for innovative approaches to redeployment and work practices [2]. There was documented variation in the redeployment processes across different hospital sites, with some describing them as voluntary while others as mandatory [3]. Regardless of their prior expertise or education, staff members either took on new responsibilities or performed equivalent tasks in other settings to aid in patient care and team efforts throughout the epidemic [4].

#### COVID-19 impacted Emergency Department visits in 2020 therefore skewed estimates

In addition, another significant limitation was that the COVID-19 pandemic in 2020 lowered the number of visits to emergency departments, which means that the model’s baseline utilization data did not represent steady or regular demand for emergency care and probably understated real physician need. During the COVID-19 pandemic, there was a 20% decrease in the average daily emergency department visits compared to the same period in 2019 [5]. In the early stages of public health initiatives aimed at reducing the transmission of the virus and preparing for potential increases in COVID-19 cases, the recommendation was to limit unnecessary healthcare consumption [6]. Additionally, several public health-related regulations were put into place to curb the spread of the virus, such as the stay-at-home order, company closures, and the requirement to wear masks [7]. There was a noticeable rise in patients visiting emergency departments (EDs) due to COVID-19, but health systems also reported a change in ED attendance for acute treatment that was unrelated to the virus [8]. As a result, when COVID-19 spread in 2020, the trend of ED visits shifted. Researchers examined how ED visits were affected by the COVID-19 pandemic in the US between January 1, 2019, and May 30, 2020 [9]. According to these findings, emergency department visits in the US fell 42% amid the pandemic [9]. Additionally, they showed that the most dramatic drop in emergency department visits occurred during the pandemic in the Northeast region, among female patients, and those younger than fourteen years old. Another team [10] presented an update on the previous [9] report; They compared emergency department visits in the US from December 20, 2020, to January 16, 2021, after the pandemic, and from December 15, 2019, to January 11, 2020, before the pandemic. According to the report, from 2020 to 2021, emergency department visits were 25% lower compared to the same months in 2020 [10]. Emergency department (ED) visit numbers were distorted in 2020 due to a drop in patients, which impacted previous predictions.

## Post-Pandemic Workforce Trends (2020–2025)

### Emergency Departments (EDs) Visit Volumes

**Figure 1:**
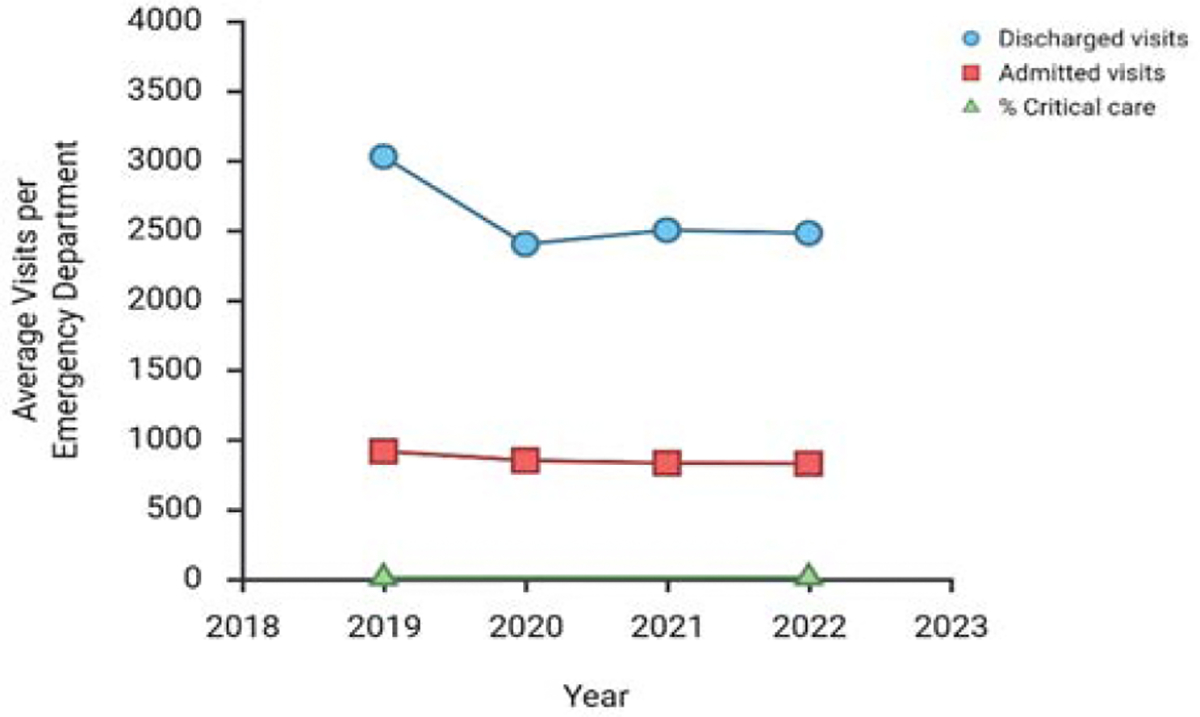
Sharp decline in the volume of emergency department visits during the COVID-19 pandemic and only partially recovered by 2022.

**Figure 2:**
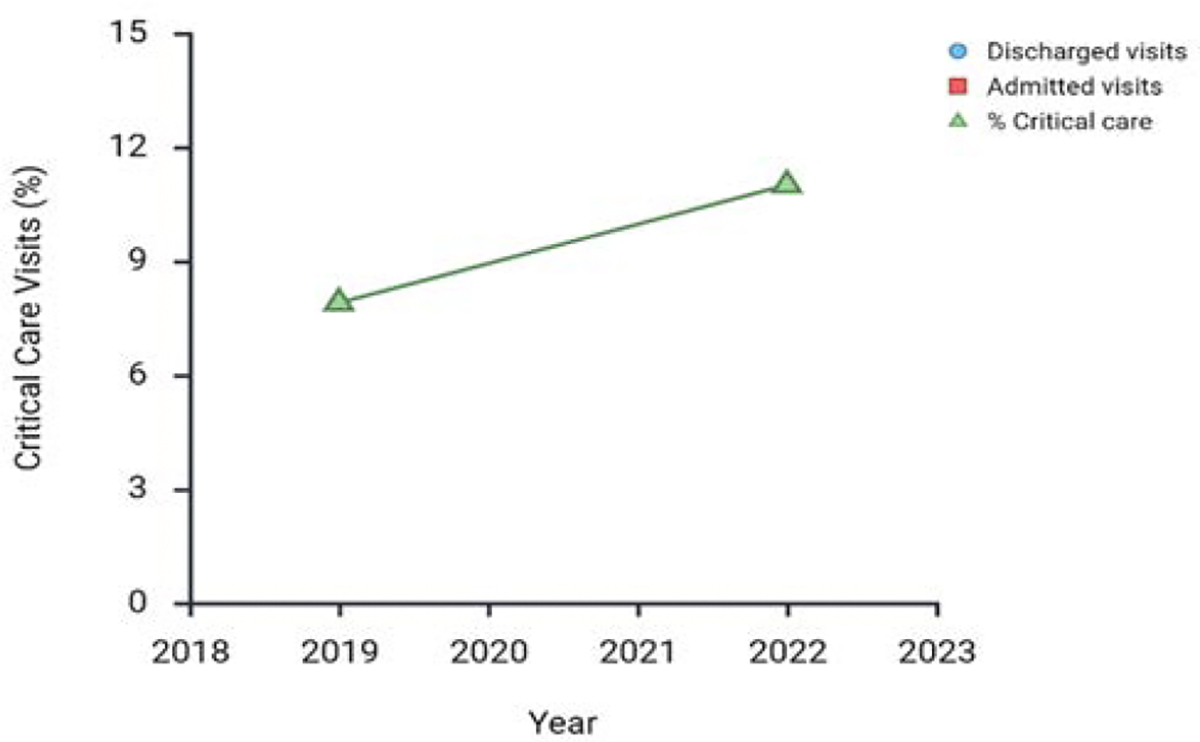
Percentage of visits requiring critical care services over the four-year study period between the years 2019 to 2022: Despite fluctuations in overall department volume, the proportion of critical care cases rose from 7.9% in the year 2019 to 11% by the year 2022.

Emergency department visit volumes declined sharply during the early stages of the pandemic and have only partially recovered. By 2022, average monthly ED volumes had increased but remained below 2019 levels (Figure 1). Although total visit counts declined, the proportion of visits categorized as critical care increased from 7.9% in 2019 to 11.0% in 2022 (Figure 2). Between 2019 and 2022, the proportion of visits billed under CPT code 99285 increased from 35.4% to 40.0%, despite an overall reduction in visit volume. Length of stay for admitted patients increased substantially, with a 32% increase in median duration and a 47% increase in upper-quartile duration compared with 2019. Patient discharge rates also increased markedly [11]. Longitudinal data from 2017 to 2023 demonstrate that emergency department visits were increasing at an annual rate of 3.4% prior to March 2020. Following relaxation of public health restrictions in 2021, visit volumes approached pre-pandemic levels before plateauing [12]. Post-pandemic demographic trends varied, with sustained increases among older age groups and declines among the least socioeconomically disadvantaged populations [12].

### Emergency Medicine Physician Workforce

**Figure 3:**
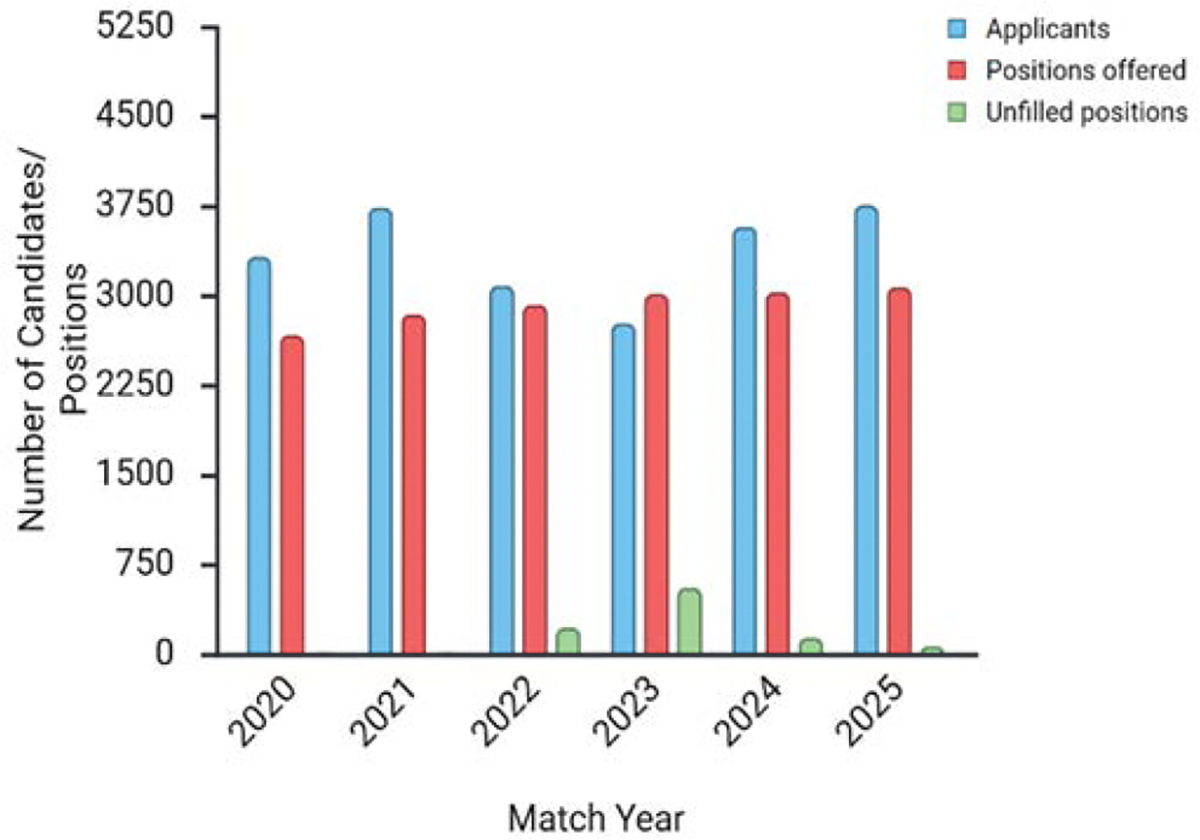
Volatility in the Emergency Medicine Residency pipeline showing fluctuations between the years 2020–2025 in the supply of emergency medicine physicians. There has been a consistent growth in the number of emergency medicine clinical residency positions offered. However, in 2023 and 2024, there was a noticeable increase in the number of unfilled positions, with 554 and 135 vacancies.

From 2001 to 2021, the number of candidates to emergency medicine (EM) grew every year except for the year 2014, reaching a peak of 3,734 in 2021 [13]. As a matter of fact, EM was so competitive that it had less than 30 unpaired residency seats per year for more than ten years prior to 2022 [14]. With a precipitous 17.5% drop from 3,734 in 2021 to 3,081 in 2022, the National Resident Matching Program (NRMP) Emergency Medicine Match left 219 openings unfilled [14]. At its peak in 2023, when 2,765 applications were received, the trend continued with a further 10.3 percent decline. Out of 3,010 open positions, 554 (18%) went unmet before the Supplemental Offer and Acceptance Program, leaving 132 (46%) of EM programs empty [13]. For the first time in decades, the emergency medicine residency program was facing a severe shortage of candidates. The 2024 match was met with cautious optimism. An increase of 29.3 percent from 2023 saw 3,574 applications for 3,026 openings in 292 EM programs. Although there was a steady increase in the number of positions (2,665 in 2020, 2,840 in 2021, 2,921 in 2022, and 3,010 in 2023), the pace of expansion has slowed for 2024, with the addition of 16 positions [13, 14]. The 2025 EM Match was much better than the previous year. The number of applications increased from 3,574 in 2024 to 3,753 in 2025, with 3,068 opportunities offered across 292 programs. Much lower than the 554 and 135 unfilled spots in the 2023 and 2024 Match, respectively, the great majority (3,003, 97.9%) of the positions were filled, leaving 65 empty slots [13]. There has been a steady decline in the number of open positions since 2023’s peak, as shown in the 2025 NRMP EM Match (Figure 3).

Early studies indicated that health care employment had not entirely recovered by the end of 2022, with results differing by location. By 2020–2022, physician office employment had fully recovered, while long-term care facility staffing was 10.5% lower than pre-pandemic levels. Employment in the health care industry fell 6.9% between the fourth quarter of 2019 and the second quarter of 2020 compared to levels projected before the pandemic [15]. Following this, the total number of jobs in the health care industry rose to 24,401,427 in the third quarter of 2024, just 0.2% below than expected [15]. On the other hand, non-health care employment reached 127,548,688 jobs in the fourth quarter of 2019, and dropped even more in the second quarter of 2020, falling 11.4% below projections. Compared to health care employment, non-health care employment recovered at a slower rate in the third quarter of 2024, falling 2.9% short of predictions [15].

## Comparative Analysis: 2020 Projection vs. Current Reality

### Workforce Supply

The participation rate in EM training has maintained a steady level of above 85% [16]. An alternate pathway for obtaining EM training has been created [17]. The emergency department now employs more physician associates, advanced practice nurses, and clinical nurse specialists [17]. Despite these gains, the increasing number of people visiting emergency departments is outpacing the rise in the workforce [18]. Although there has been some improvement in the workforce situation, it is still far from resolved due to issues such as high turnover rates in training programs and the early retirement of accomplished doctors [18]. The emergency residency program at Christus Spohn Hospital in Corpus Christi, Texas, is going to be completely shut down due to financial issues and an excess of emergency residency spots [19]. The pronouncement has upset medics and patients all over Corpus Christi and Texas, people who are concerned about the possibility of a neglected neighborhood losing access to a supply of doctors because it is the sole emergency medicine residency in the state of South Texas [19].

### Workforce Demand

Emergency department visits decreased at disproportionate rates for patients under the age of 18 (−60.1%) and over the age of 65 (−41.3%), females (−40.2%), Whites (37.8%) and Asians (40.2%), patients with Medicare (−40.8%), patients with other insurance (74.1%; for example, workers’ compensation, no-fault, liability), patients who were ambulatory (38.1%), and patients who left before evaluation or discharge (75.6%) [8]. Patients aged 65 and up, as well as Medicare participants, experienced substantial proportional declines in the frequency of their visits. The number of visits by women, children, and people of specific races decreased at a much faster rate than by any other demographic. The percentage of patients who departed prior to assessment or release also decreased significantly [8]. However, the most worrisome aspect of this data is the general decrease in patients seen for serious and perhaps fatal illnesses that are unconnected to COVID-19 [8]. Researchers found what one would expect: a significant drop in the number of people presenting with less serious symptoms like back pain and generalized discomfort. Similarly to what has been observed in other studies on myocardial infarction presentations, researchers also discovered surprisingly disproportionate drops in visits for significantly more serious and urgent diseases like syncope, cerebrovascular accidents, and urolithiasis [20,21].

## Discussion

The 2020 emergency medicine workforce projection was built on assumptions that, while reasonable under pre-pandemic conditions, they have since proven fragile when tested against the realities of the COVID-19 era and its aftermath. Chief among these assumptions was a relatively low annual emergency physician attrition rate of approximately 3% [1]. Post-pandemic data, however, consistently demonstrate substantially higher attrition, with overall annual rates ranging from 5.3% to 5.7% and permanent attrition estimated between 3.8% and 4.9% [22]. This discrepancy alone has profound implications for the validity of the projected physician surplus. More recent modeling indicates that increasing the assumed attrition rate by even a single percentage point reduces the projected surplus from nearly 8,000 physicians to approximately 2,486 clinicians [22]. Given that contemporary attrition rates exceed the original assumption by more than two percentage points, it is highly likely that the 2020 projection substantially overestimated future workforce supply. Attrition in emergency medicine appears to be driven not only by retirement but also by burnout, dissatisfaction with evolving practice conditions, increasing administrative burden, and shifts toward non-clinical or non-traditional roles—factors that were not adequately captured in earlier forecasting models. Beyond attrition, the 2020 projection relied heavily on utilization-based demand estimates derived from a period of unprecedented disruption in emergency department operations. Emergency department visit volumes in 2020 were intentionally suppressed through public health messaging, regulatory restrictions, and widespread behavioral changes in health care–seeking behavior [5–10]. Although these measures were necessary to mitigate viral transmission, they rendered utilization data from this period fundamentally unrepresentative of baseline or future demand. As a result, demand estimates anchored in pandemic-era utilization likely underestimated true clinical need and distorted workforce balance calculations. Importantly, post-pandemic emergency department utilization has not simply returned to pre-2020 norms but has instead evolved in complexity and acuity. While total visit volumes have remained below 2019 levels in many settings, the proportion of high-acuity and critical care cases has increased substantially [11]. Rising rates of prolonged length of stay, higher billing acuity codes, and increased admission times suggest that emergency departments care for fewer patients overall but with significantly greater resource intensity. Workforce models that equate demand solely with visit counts fail to account for this qualitative shift in clinical burden and may therefore underestimate physician workload and staffing needs. The uneven recovery of emergency department utilization across demographic groups further highlights the limitations of utilization-based forecasting. Disproportionate declines in visits among pediatric patients, older adults, women, Medicare beneficiaries, and ambulatory patients during the pandemic were accompanied by alarming reductions in presentations for serious non-COVID conditions, including myocardial infarction, stroke, syncope, and urolithiasis [8,20,21]. These findings raise concern that deferred or avoided care during the pandemic may contribute to delayed presentations, increased disease severity, and future surges in demand—none of which are adequately incorporated into static workforce projections. Although participation in emergency medicine training programs has remained relatively robust, workforce sustainability remains threatened by structural and systemic factors. The expansion of advanced practice provider roles and alternative training pathways has increased staffing flexibility [17], yet this has not translated into long-term workforce stabilization. Emergency department visit growth continues to outpace clinician workforce expansion, while turnover among both trainees and experienced physicians erodes effective supply [18]. The closure of emergency medicine residency programs, such as the program at Christus Spohn Hospital in Corpus Christi, underscores the sensitivity of the workforce ecosystem and challenges assumptions that supply will continue to increase in a linear and predictable fashion [19]. These findings suggest that workforce projections based primarily on headcount metrics are insufficient to capture the dynamic nature of emergency medicine practice. Factors such as workforce morale, practice environment, corporate staffing models, and regional variability in access to care play critical roles in shaping workforce stability but are difficult to quantify within traditional forecasting frameworks. The pandemic exposed the vulnerability of emergency medicine staffing to external stressors and highlighted the importance of resilience, adaptability, and retention in workforce planning. Taken together, post-pandemic evidence indicates that the emergency medicine workforce is more fragile than previously projected. Rather than confirming a durable oversupply, current trends suggest a workforce characterized by heightened attrition, increased clinical complexity, and susceptibility to future disruptions. Workforce models that assume stable utilization, low attrition, and predictable supply growth risk systematically underestimating demand while overstating surplus. Future projections must incorporate updated attrition data, account for changes in patient acuity and care complexity, and acknowledge the structural shifts reshaping emergency medicine practice.

## Conclusions

Emergency department utilization in 2020 was artificially suppressed, staffing models were temporarily restructured, and subsequent years have been characterized by increased patient acuity, operational strain, and higher-than-anticipated physician attrition. Together, these findings indicate that workforce models grounded in pandemic-era utilization data systematically underestimate demand and exaggerate projected physician surplus. Rather than confirming oversupply, post-pandemic trends suggest a more fragile emergency medicine workforce marked by sustained attrition and vulnerability to stressors. Based on post-pandemic utilization trends, rising patient acuity, prolonged emergency department length of stay, and sustained attrition rates exceeding those assumed in prior models, it is reasonable to project that demand for emergency physicians will remain stable or increase modestly between 2030 and 2040, rather than decline. Even in the absence of a full return to pre-pandemic visit volumes, the growing complexity of emergency care, driven by an aging population, delayed presentations of chronic and acute disease, and increased boarding and throughput challenges, suggests that clinical workload per physician is likely to increase, thereby maintaining or expanding demand for emergency physicians during this period. Future workforce projections must incorporate updated attrition estimates and account for structural shifts in emergency care delivery.
